# Enhancing Antioxidant and Flavor of Xuanwei Ham Bone Hydrolysates via Ultrasound and Microwave Pretreatment: A Backpropagation Artificial Neural Network Model Prediction

**DOI:** 10.3390/molecules31010188

**Published:** 2026-01-04

**Authors:** Xin Chen, Xianchao Feng, Xingwei Wang, Nianwen Zhang, Yuxia Jin, Jianxin Cao, Xuejiao Wang, Chaofan Guo

**Affiliations:** 1 Faculty of Food Science and Engineering, Kunming University of Science and Technology, Kunming 650500, China; chenxin139144@outlook.com (X.C.); wangxingwei818@hotmail.com (X.W.); z931544812@163.com (N.Z.); 18109733690@163.com (Y.J.); jxcao321@hotmail.com (J.C.); 2 College of Food Science and Engineering, Northwest A & F University, Xianyang 712100, China; xcfeng@nwafu.edu.cn

**Keywords:** Xuanwei ham bone, GC-MS, aroma analysis, electronic nose analysis, antioxidant capacity

## Abstract

This study aimed to produce the hydrolysates of Xuanwei ham bone using enzymatic hydrolysis assisted by microwave and ultrasound pretreatment. A back propagation artificial neural network (BP-ANN) model was utilized to predict the optimal conditions, which involved 15 W/g bone for 15 min of ultrasound pretreatment and 5 W/g bone for 30 min of microwave pretreatment, achieving the highest degree of hydrolysis (DH). The model predicted a DH of 27.69, closely aligning with the experimentally measured actual DH of 28.33. DPPH radical scavenging and TBARS demonstrated that hydrolysates prepared by ultrasound combined microwave pretreatment (UMH) exhibited the highest antioxidant activity and significantly inhibited lipid oxidation. GC-MS analysis revealed that the UMH showed removal of bitter volatile flavor compounds, such as o-Cresol and m-Cresol, the retention of aromatic volatile compounds, such as 2-pentylfuran, formation of new aromatic volatile compounds such as 3-methylbutanal, and the reduction in certain aldehyde and ketone compounds. Pearson correlation analysis elucidated that the reduction in aldehyde and ketone compounds was positively linked to the enhanced antioxidant capacity of UMH. The results obtained hold substantial significance for enhancing the added value of Xuanwei ham within the food industry.

## 1. Introduction

Xuanwei ham, celebrated for its distinctive and enjoyable flavor, is a prominent traditional Chinese dry-cured ham [1]. During the processing and sales of Xuanwei ham, a significant amount of bone waste is generated, leading to resource wastage and environmental costs. Bones are a crucial source of collagen protein [2]. Numerous studies have demonstrated that valuable components can be extracted from bones through enzymatic hydrolysis, thereby enhancing the added value of dry-cured ham [3,4,5,6,7].

Conventional enzymatic hydrolysis methods often suffer from prolonged processing times, high energy consumption, low enzyme efficiency, and suboptimal substrate conversion and hydrolysis rates [8]. Utilizing new pretreatment techniques, which involve chemical and physical, can significantly enhance the degree of hydrolysis (DH) and improve the functional properties of the hydrolysates [3]. Liu et al. [9] employed ultrasound-assisted enzymatic hydrolysis to enhance the hydrolysis rate of mung bean protein and significantly improved the antioxidant capacity of the hydrolysates. Zheng et al. [4] reported that combining ultrasound with microwave pretreatment enhanced the DH in beef bone hydrolysates and generated more flavor compounds. Additionally, microwave-ultrasound pretreatment significantly enhanced the antioxidant properties of bovine bone hydrolysates compared to ultrasound pretreatment alone. During enzymatic hydrolysis, ultrasound was beneficial as it unfolds protein structures and enhanced the affinity between enzymes and proteins due to the high temperatures, pressures, and shear forces it generated [10]. Similarly, during enzymatic hydrolysis, molecular motion was elicited by microwave energy through ion conduction and dipole rotation [11]. Moreover, Habinshuti et al. [12] reported that employing microwave pretreatment following ultrasound can further enhance hydrolysis efficiency, as microwave electromagnetism may accelerate the structural changes in protein molecules induced by ultrasound treatment. However, the technical parameters of pretreatment and the DH are key factors influencing the antioxidant properties of enzymatic hydrolysates [13]. Zhou et al. [7] reported in their review that microwave and ultrasound pretreatments with different power ratios and duration increased in DH by 8–165%. Therefore, balancing the output power and duration of pretreatment to provide an optimal enzymatic environment is crucial for enhancing the antioxidant capacity of enzymatic hydrolysates.

An artificial neural network (ANN) is a computational framework modeled after the neural networks in the human brain [14]. ANN can independently learn, adapt to new conditions, and handle faults, enabling them to map the nonlinear structures of complex dynamic phenomena [15]. Currently, various ANNs are in use, among which back-propagation ANN (BP-ANN) are particularly appreciated by many developers for their effective nonlinear mapping, self-learning capacities, adaptability, and generalized fault tolerance [16]. BP-ANN enables error feedback throughout the network and the continuous optimization of weights and biases, serving as a standard technique to minimize the loss function in ANN [17]. In recent years, numerous studies have reported the use of ANN to optimize enzymatic hydrolysis process parameters, including protease concentration, hydrolysis duration, and solid-to-liquid ratio [18,19]. However, there is limited literature on optimizing emerging technologies that assist enzymatic hydrolysis, such as ultrasound and microwave. Excessive use of ultrasound and microwave power may lead to over-degradation of protein structures and resource wastage, while insufficient application might not yield significant effects.

This study aimed to elucidate how ultrasound and microwave pretreatments affect the DH, antioxidant capacity, and flavor attributes of Xuanwei ham bone hydrolysates. In parallel, we develop and validate a BP-ANN to predict DH from controllable pretreatment parameters. By integrating multi-physics pretreatment with data-driven modeling, the study provides a practical basis and predictive tool for valorizing ham bone by-products and guiding parameter selection in industrial production, with the potential to reduce processing time and energy consumption. Scientifically, it advances understanding of ultrasound–microwave synergies on protein substrate structure and hydrolysis outcomes and contributes a validated modeling framework and dataset to support future optimization and scale-up in food protein valorization.

## 2. Results and Discussion

### 2.1. BP-ANN Model Development

#### 2.1.1. Optimization of the Hidden Nodes Number

**Figure 1:**
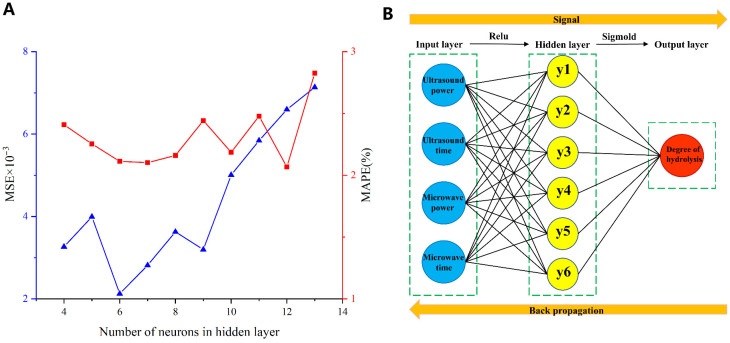
(**A**) BP-ANN fitting effects of different neurons; (**B**) BP-ANN structure diagram.

In the design of the BP-ANN, it is essential to determine the optimal number of neurons in the hidden layer [20]. To counteract the effects of random weight initialization by the MATLAB software, each topology was repeated 10 times, and both mean MSE and MAPE were recorded. Figure 1A illustrates the relationship between network MSE, MAPE, and the number of nodes in the hidden layer. MSE quantifies model accuracy in regression tasks by calculating the square of the discrepancy between predicted and actual values [21]. A lower MSE suggests a closer approximation to true values. MAPE, which ranges from 0 to infinity, indicates model precision [22]. A MAPE of 0% denotes a perfect model, while values above 100% suggest a less effective model [14]. The results in our study showed that the MAPE for all hidden layer nodes varied between 2% and 3%, demonstrating excellent model fit. The smallest values were observed when the number of hidden layer nodes was 6, 7, or 12. Notably, the network achieved the minimum MSE with 6 nodes in the hidden layer. Consequently, this configuration of 6 neurons in the hidden layer was chosen for further network development. As shown in Figure 1B, a 4-6-1 fully connected BP-ANN model was constructed.

#### 2.1.2. Training, Validation and Testing of the Model

**Figure 2:**
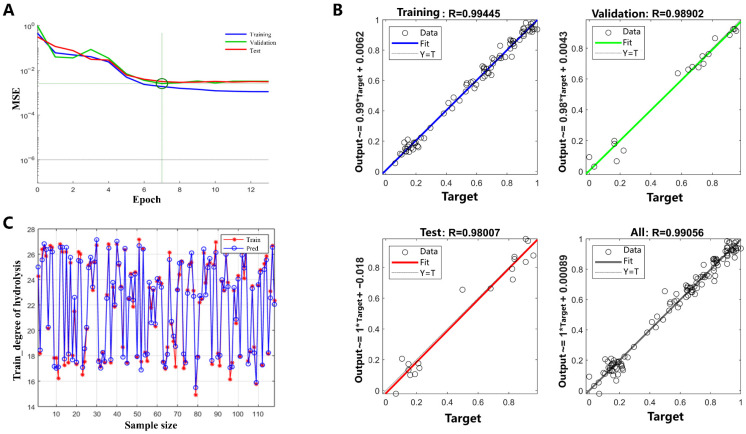
(**A**) Network performance during training; (**B**) Scatter plots of predicted values from the ANN model versus experimental values for training, validation, testing, and all datasets; (**C**) Prediction performance of training set of DH (* denotes multiplication).

The 169 datasets were allocated into training (70%), validation (15%), and testing (15%) subsets. Training convergence occurred at 7 epochs, where network performance (MSE) stabilized, as shown in Figure 2A, indicating optimal training performance. The overall MSE across all datasets was recorded at 0.00215. Figure 2C illustrates the comparison between the predicted outcomes of the model on the training set and the actual values. The observed differences between the predicted (Pred) and actual (True) results were minimal, demonstrating that the model exhibits high prediction accuracy and strong generalization capabilities without any signs of overfitting. Figure 2B presents scatter regression plots comparing the BP-ANN predicted values with the experimental data. According to Figure 2B, the training, validation, and testing subsets demonstrated strong linear correlations, with correlation coefficients of 0.9945, 0.9890, and 0.9801, respectively. The model of BP-ANN line closely matched the ideal fit line (45-degree diagonal), with an overall correlation coefficient of 0.9906, showcasing the model excellent alignment with the experimental observations. Therefore, the developed BP-ANN model can predict the effects of ultrasound and microwave pretreatment on enzymatic hydrolysis efficiency with high accuracy.

#### 2.1.3. Contribution of Each Input Variable on DH

Following the development of the model, connection weights between layers were assessed (Table 1) to determine the influence of each input variable on the DH. The relative importance of four input variables on the output was quantified using Equation (8). Ultrasound power was identified as the predominant factor in the enzymatic hydrolysis process, accounting for 50.35% of its relative importance, highlighting the crucial role of ultrasonic pretreatment in the hydrolysis of Xuanwei ham bones. This effect may be attributed to the fact that ultrasound could generate oscillating compression and rarefaction cycles in the bone hydrolysates, leading to acoustic cavitation and turbulence, which disrupt the structural integrity of substrate proteins, thereby enhancing the enzymatic process [23].

Furthermore, Ayim et al. [24] indicated that ultrasonic pretreatment can modify the kinetics of enzymatic reactions and enhance the enzymatic hydrolysis efficiency by altering substrate structures. Ultrasound treatment could attenuate hydrogen bonds [25], Van der Waals forces [26], and hydrophobic interactions [27] existed in inter- or intramolecular proteins, which loosened the protein structure and facilitated enzymatic hydrolysis. Furthermore, ultrasound treatment may also change the surface morphology of substrates, increasing the contact area between the enzyme and substrate [28]. Abdualrahman et al. [29] reported that ultrasonic treatment modifies the thermodynamic parameters of the enzymatic process. The reductions in activation energy (Ea) and enthalpy change (ΔH) induced by ultrasonic pretreatment lowered the energy barriers, enhanced reaction efficiency, and adapted enzyme-substrate complexes to more active states. The decrease in ΔH suggested that ultrasound pretreatment may modify protein structures by disrupting hydrophobic interactions and stabilizing enzyme-protein interactions, facilitating the oxidative adaptation of amino acid residues and optimizing protein configuration [30].

It was followed by microwave power, with relative importance values of 20.43%. The effects of the two pretreatment times on the DH of Xuanwei ham bone enzymatic hydrolysates had similar relative importance, and the ultrasound time (15.36%) was slightly higher than the microwave time (13.86%). Compared with ultrasonic pretreatment, high-power microwave (15 W/g and 20 W/g)pretreatment did not significantly alter the DH, which was consistent with earlier findings by Hall and Liceaga [31]. Subsequently, under the conditions of low-power microwave pretreatment at 5 W/g and 10 W/g, a significant increase in the DH was observed as the pretreatment duration extended. Notably, at 5 W/g, a single low-power microwave pretreatment for 20 min exhibited effects comparable to those of a single ultrasound pretreatment at 5 W/g for 15 min, with DH of 17.36% and 17.23%, respectively. This variation is attributed to the different of food ingredients, protease properties and their interactions with the substrates. The gentle, sustained heating from low-power microwave treatment promotes a more uniform thermal distribution and gradual breakdown of substrate structures, thereby improving enzymatic accessibility and effectiveness compared to the rapid and localized heating typical of high-power microwave treatment. Li et al. [32] also reported that low-power, prolonged microwave exposure (300 W for 30 min) enhanced the DH and reduced the hydrolysis time.

#### 2.1.4. Prediction of DH by Each Input Variable

In accordance with the discussions detailed in Section 2.1.3, we adjusted the microwave parameters to lower power levels (5, 7 W/g bone) and increased the duration (20, 30 min), while employing high-power ultrasound pretreatment (15, 20 W/g bone) for a duration of 15 min. The BP-ANN model, optimized by Section 2.1.2, was utilized to predict the DH. As indicated in Table 2, the predicted DH values under various pretreatment conditions were consistently lower than the experimentally measured values. The discrepancy likely resulted from inadequate coverage of prolonged microwave preprocessing in the training dataset, leading to poor model generalization. Nonetheless, the overall trend in DH predicted by the BP-ANN closely reflected the actual experimental outcomes. Notably, the most significant increase in DH was observed when employing an ultrasonic power of 15 W/g bone for 15 min, paired with low microwave power (5, 7 W/g bone) and extended duration (30 min). Additionally, it was noted that increasing the ultrasound intensity to 20 W/g bone did not further enhance the DH of the Xuanwei ham bone hydrolysates, likely due to the high-intensity ultrasound excessively damaging the protein structure and reducing the availability of effective enzyme cleavage sites [33]. Therefore, considering both cost and efficiency in actual processing, optimal parameters were established as 15 W/g bone and 15 min for ultrasound, along with 5 W/g bone for microwave power, and a duration of 30 min for the subsequent preparation of Xuanwei ham bone enzymatic hydrolysates. Under these conditions, the highest enzymatic degree was achieved, with the BP-ANN model predicting a DH of 27.69, while the experimentally measured actual DH was 28.33 ± 0.18.

### 2.2. Antioxidant Capacity

#### 2.2.1. Changes in DPPH Free Radical Scavenging Assay

**Figure 3:**
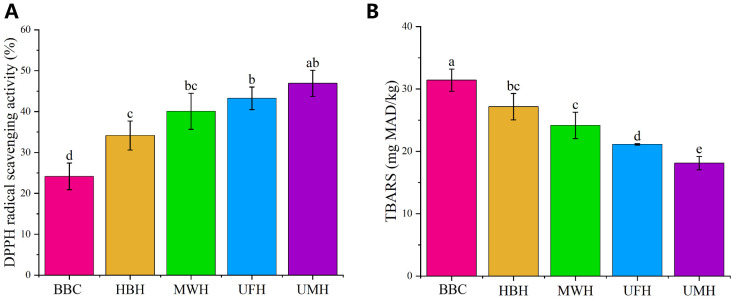
Antioxidant capacity of Xuanwei ham bone hydrolysates with different pretreatments, (**A**) (DPPH radical scavenging ability), (**B**) (TBARS). (BBC: Bone broth control; HBH: Ham bone hydrolysates; MWH: Microwave pretreatment hydrolysates; UFH: Ultrasound pretreatment hydrolysates; UMH: Ultrasound combined microwave pretreatment hydrolysates). Different letters indicate significant differences (*p* < 0.05).

The antioxidant capacity of Xuanwei ham bonehydrolysates was assessed using DPPH radical scavenging activity. As shown in Figure 3, the DPPH free radical scavenging activity of hydrolysates under different pretreatments varies significantly. Compared to bone broth control (BBC), ham bone hydrolysates (HBH) exhibited higher DPPH radical scavenging activity (*p* < 0.05), indicating that enzymatic hydrolysis enhanced the antioxidant capacity of the hydrolysates. This enhancement is attributed to proteases cleaving protein macromolecules during enzymatic hydrolysis [34]. This process results in smaller peptide fragments and amino acids with antioxidant properties, such as cysteine, lysine, and arginine [35,36]. This finding was consistent with the work of Xu et al. [37], who reported similar antioxidant activities in hydrolysates obtained from soy protein digested with alkaline protease.

Compared to HBH, the pretreated hydrolysates showed higher DPPH radical scavenging activities, especially the hydrolysates prepared by ultrasound pretreatment (UFH) (*p* < 0.05). This suggested that both microwave and ultrasonic pretreatments assisting enzymatic hydrolysis can further enhance the antioxidant capacity of the hydrolysates, which was consistent with the reports by Liu et al. [9] and Ketnawa and Liceaga [38]. Microwave pretreatment inducing protein conformational changes and protein unfolding, which increases hydrolysis and generates more low molecular weight bioactive peptides, thus enhancing overall antioxidant capacity [39]. The shearing and cavitation effects of ultrasound induced protein unfolding and exposed more hydrophobic domains, facilitating the production of more hydrolytic products [40]. Notably, UMH showed the highest DPPH radical scavenging activity and the lowest TBARS values (*p* < 0.05) compared to hydrolysates prepared solely by microwave (MWH) or ultrasound (UFH). This suggested that microwave and ultrasound had a synergistic effect and further enhanced the antioxidant properties of the hydrolysates.

#### 2.2.2. Changes in TBARS

TBARS value serves as a conventional metric for evaluating the extent of secondary lipid oxidation [41]. As shown in Figure 3, the TBARS of hydrolysates under different pretreatments showed an opposite trend to that of DPPH free radical scavenging activity. Enzymatic hydrolysis significantly reduced the TBARS in the hydrolysates (*p* < 0.05). Although MWH further decreased TBARS, this reduction was not statistically significant (*p* > 0.05). This might be attributed to excessive lipid oxidation in the hydrolysates due to prolonged microwave exposure (30 min). The UMH exhibited the lowest TBARS (*p* < 0.05), consistent with the DPPH assay results. This suggests that the incorporation of ultrasound can compensate for the shortcomings of prolonged microwave exposure, and the synergistic effect of these combined pretreatments effectively inhibits lipid oxidation in the hydrolysis.

In summary, among all treatments, UMH exhibited the strongest antioxidant capacity and effectively inhibited lipid oxidation in the hydrolysates. This was not only attributed to its high DH, which generated more antioxidant-rich hydrolysates, but also to the synergistic non-thermal processing techniques of ultrasound combined with microwave pretreatment.

### 2.3. Aroma Profiles

#### 2.3.1. HS–SPME/GC–MS Analysis

**Figure 4:**
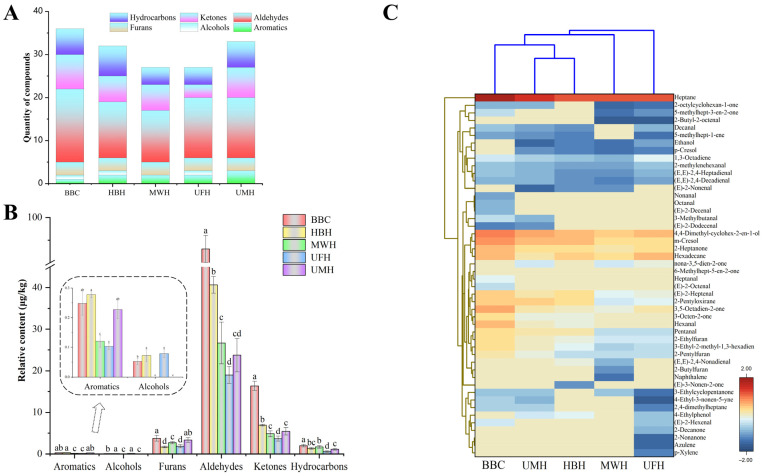
(**A**) Quantity of volatile compound category, (**B**) relative content of volatile compound classes, and (**C**) heat map clustering of volatile compounds in Xuanwei ham bone enzymatic hydrolysates under different pretreatments examined by GC-MS (BBC: Bone broth control; HBH: Ham bone hydrolysates; MWH: Microwave pretreatment hydrolysates; UFH: Ultrasound pretreatment hydrolysates; UMH: Ultrasound combined microwave pretreatment hydrolysates). Different letters indicate significant differences (*p* < 0.05), see also Supplementary Materials.

To more comprehensively analyze the impact of ultrasound and microwave pretreatments on the volatile compounds in ham bone hydrolysates, we conducted qualitative and quantitative analyses of the aroma compounds under different pretreatments by using HS-SPME/GC-MS. A total of 48 volatile compounds were identified from the hydrolysates, including 6 aromatic compounds, 2 alcohols, 3 furans, 18 aldehydes, 11 ketones, and 8 hydrocarbons. Their specific categories were shown in the heat map Figure 4C. As shown in Figure 4A, the highest composition of volatile compounds, amounting to 36 types, was detected in the untreated BBC. In contrast, MWH and UFH exhibited the fewest types of volatile compounds, which was inconsistent with previous reports of Li et al. [42]. They only observed a difference in the composition of volatile compounds between the ultrasound group and the control, but the overall amount was not reduced. This inconsistency may be due to variations in enzyme specificity or differences in the power and time of the pretreatments. Notably, UMH significantly increased the variety of volatile compounds, suggesting that this synergistic effect may enhance the formation of flavor compounds.

As illustrated in Figure 4B, after enzymatic treatment, except for furans, aromatics, and alcohols, the remaining volatile compounds in the hydrolysates showed a relative decrease in concentration, particularly the ketones, aldehydes, and hydrocarbons. Prolonged enzymatic reactions may affect the stability and presence of volatile compounds in aqueous solutions. Notably, the relative content of aldehydes and ketones in the pretreated hydrolysates was significantly lower than in the untreated samples (*p* < 0.05). The formation of ketones and aldehydes is predominantly through lipid oxidation processes, such as the oxidation of unsaturated fatty acids like linoleic and linolenic acids. We speculated that this may be because the ultrasound and microwave pretreatment have inhibited lipid oxidation, thereby reducing the generation of aldehydes and ketones, which was consistent with TBARS results (Figure 3). The lowest levels of aldehydes and ketones were detected in UFH, which can also be attributed to the mechanical vibrations and cavitation effects of ultrasound [4]. In contrast, UMH showed a slight increase in the levels of aldehydes and ketones, indicating that combined microwave pretreatment may alleviate this disadvantage. Additionally, when ultrasonic and microwave treatments were integrated, the concentrations of furan compounds approximated those in the original bone broth, especially for 2-ethylfuran (BBC: 0.13 ± 0.02, UMH: 0.11 ± 0.02) and 2-butylfuran (BBC: 0.19 ± 0.03, UMH: 0.17 ± 0.01). These two volatile compounds are described as having strong roasted and smoky flavors [43]. Overall, the synergistic application of ultrasound and microwave treatment not only alleviates the adverse effects noted with their individual use but also promotes the generation of specific volatile compounds.

Table 3 highlights key volatile compounds associated with the hydrolysates, with odor activity values (OAV) greater than 1, totaling 24 distinct volatiles. p-Cresol and m-Cresol, which were characterized by their bitter taste [44], were detected with substantial contributions only in samples UFH and MWH, and were absent in the UMH samples. 2-Pentylfuran, noted for its green bean and buttery aroma [3], contributed significantly to the aroma profile in both the BBC and UMH samples. Aldehydes are predominant in Table 3 with 17 types due to their generally lower odor thresholds. Most of the aldehydes presented appealing flavors, such as the leafy aroma of Hexanal, the toasted bread scent of (E, E)-2,4-Decadienal, the nutty fragrance of (E)-2-Heptenal, and the citrusy smell of Nonanal [45]. Regrettably, high concentrations of these aldehydes were not detected in the UMH-treated samples, except for (E, E)-2,4-Nonadienal and 3-Methylbutanal. Ketones also exhibited similar trends.

#### 2.3.2. E-Nose Analysis

**Figure 5:**
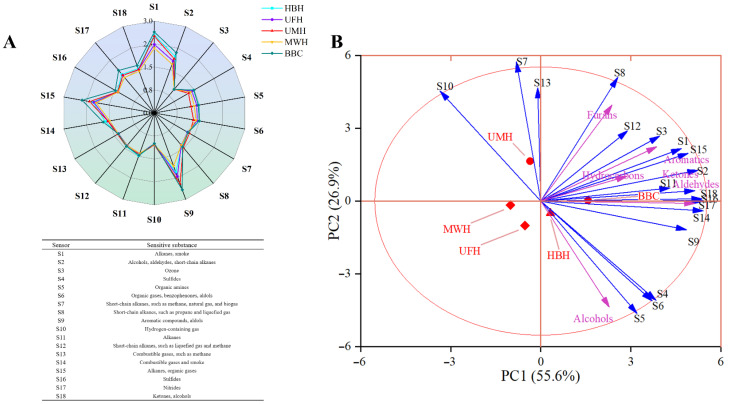
(**A**) Radar chart of electronic nose analysis for Xuanwei ham bone enzymatic hydrolysates with different pretreatments; (**B**) PCA of aroma and sensory scores (BBC: Bone broth control; HBH: Ham bone hydrolysates; MWH: Microwave pretreatment hydrolysates; UFH: Ultrasound pretreatment hydrolysates; UMH: Ultrasound combined microwave pretreatment hydrolysates).

The radar chart depicted the sensor responses to various volatile compounds in Xuanwei ham bone hydrolysates under distinct treatment conditions (Figure 5A). Eighteen sensors detected fourteen different classes of volatile substances, with the magnitude of the response values indicating the concentration of these compounds. Sensors S1 (Alkanes), S2 (Alcohols, aldehydes, short-chain alkanes), S9 (Aromatic compounds, aldols), and S15 (Alkanes) recorded higher response values, indicating that the volatile compounds corresponding to these sensors have higher contents in bone hydrolysates. Furthermore, variations in the response values at sensors S1, S2, S9, and S15 among different samples. BBC had the highest response value among S1, S2, S9, and S15, followed by HBH and UMH components, which was consistent with the results of GC-MS. These findings suggested that both enzymatic hydrolysis and ultrasound-microwave pretreatment significantly influence the concentrations of volatile substances.

Principal component analysis (PCA) effectively reduces the dimensionality of complex data while capturing the essential information contained within the samples. As illustrated in Figure 5B, PC1 accounts for 55.6% and PC2 for 26.9% of the variance, cumulatively explaining 82.5% of the data. A clear separation between the UMH and the rest of the components was observed along PC2. The volatile components identified by GC-MS, prominently aldehydes, ketones, aromatics, and hydrocarbons, show a strong correlation with the odors detected by the E-Nose.

### 2.4. Correlation Between Basic Properties and Aroma

**Figure 6:**
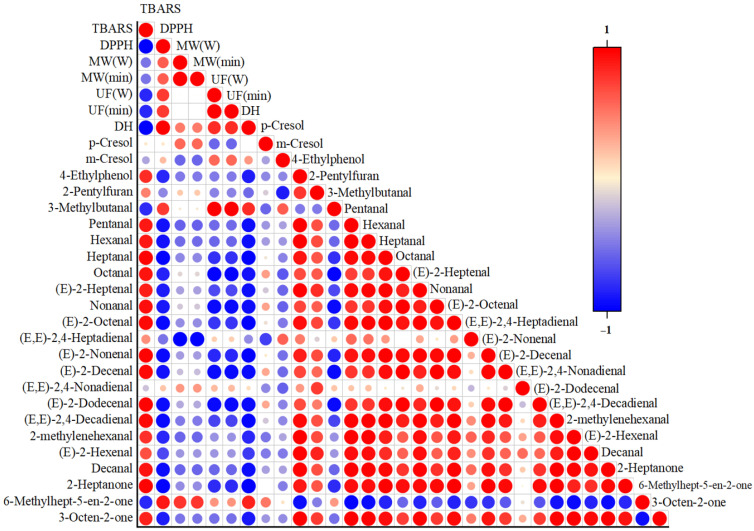
Correlation between basic properties and flavor of Xuanwei ham bone enzymatic hydrolysates.

Figure 6 presents the Pearson correlation analysis between the basic properties and aroma profiles of Xuanwei ham bone hydrolysates under different pretreatments. The ultrasound power and time, as well as microwave power and time, showed a positive correlation with the DH of Xuanwei ham bone hydrolysates, indicating that the inclusion of microwave and ultrasound pretreatment significantly enhanced hydrolysis. The DH of the hydrolysates was negatively correlated with TBARS and positively correlated with DPPH radical scavenging ability, that is, lower TBARS and higher DPPH radical scavenging capacity indicated an improvement in the antioxidant capacity of the hydrolysates. This suggested that the antioxidant capacity of the hydrolysates increased progressively during hydrolysis, which was primarily attributed to the disruption of the original macromolecular structure during the hydrolysis process, resulting in the formation of small molecular compounds endowed with antioxidant properties, such as short-chain peptides and free amino acids [46].

Additionally, we found that TBARS was significantly positively correlated with most aldehydes (except for 3-methylbutanal, (E, E)-2,4-Nonadienal), while DPPH radical scavenging ability was significantly negatively correlated with most aldehydes. This implied that as the antioxidant capacity increased, the content of aldehyde compounds decreased. Aldehyde compounds were primarily produced through lipid oxidation and amino acid degradation. 3-Methylbutanal was detected only in the UFH and UMH samples (Table 3), described as having a strong creamy or nutty flavor, primarily derived from the leucine degradation rather than lipid oxidation [47]. This outcome might be attributed to the shear forces and cavitation effects of ultrasound, which can disrupt cellular structures and accelerate the Strecker degradation of amino acids [48]. The remaining aldehydes were all associated with lipid oxidation. Oxidation of linoleic acid led to the formation of compounds, such as heptanal, (E)-2-nonenal, (E)-2-decenal, and (E, E)-2,4-decadienal [49]. From the oxidation of linolenic acid, compounds, such as (E, E)-2,4-nonadienal, were produced. (E, E)-2,4-Decadienal resulted from the decomposition of linoleic acid hydroperoxides during the initial stages of lipid oxidation and served as a crucial intermediate in furan formation [50]. Although the reasons behind the positive correlation between (E, E)-2,4-nonadienal and antioxidant capacity remain unclear, variations in concentrations of (E, E)-2,4-decadienal or (E, E)-2,4-nonadienal alone do not significantly alter the broth-like aroma profile [51].

3-Octen-2-one and 2-Heptanone, like most aldehydic compounds, exhibit trends positively correlated with TBARS and negatively correlated with DPPH, indicating an inverse relationship with the antioxidant capacity of ham bone hydrolysates. 3-octen-2-one, a secondary product of lipid oxidation, is characterized by a strong vanilla flavor [52]. 2-heptanone, a common methyl ketone and a tertiary product of lipid oxidation, can be formed through various pathways from secondary lipid oxidation products [53]. In summary, the pretreatment with ultrasound and microwave enhances the antioxidant capacity of hydrolysates, which in turn reduces lipid oxidation in the hydrolysates, thereby further decreasing the release of aldehydic and ketonic compounds. This also explains the reduction in volatile aldehydes and ketones in UMH.

## 3. Materials and Methods

### 3.1. Materials

Xuanwei ham was purchased from Xuanwei Yongjin Ham Factory in Xuanwei of Yunan Province in China. Food-grade Protamex 1.6 L and Flavourzyme 500 MG were purchased from Novozymes (Tianjin Novozymes Biotechnology Co., Ltd., Tianjin, China). All chemicals procured were of analytical grade and were employed in their original form.

### 3.2. Sample Preparation Before Enzymatic Hydrolysis

Manual deboning was performed to remove surface meat residues and fascia, and the bone marrow was scooped out. The surfaces and inner cavities of the bones were rinsed with running water to remove most of the adhering fats and salts. The samples were then placed in an oven and dried at 40 °C for 1.5 h. After drying, the samples were ground using a crusher (BJ-1000A, Deqing, Huzhou, China) and then sieved through a 100-mesh screen to obtain bone meal.

### 3.3. Preparation of Enzymatic Hydrolysates

The chopped bone powder was mixed with water in a 1:8 (*w*/*w*) ratio and stirred for 2 min. The mixture was subjected to ultrasound pretreatment using an ultrasound cell disruptor (SCIENTZ-IID, Scientz Technology Co., Ningbo, China) at 25 kHz in a pulsed mode (2 s on/3 s off). Microwave heating was conducted using a microwave device (ORW1.0S-5H, Orientmw Technology Co., Nanjing, China) in an intermittent mode (20 s on/40 s off). Ultrasound and microwave treatments were applied at power levels of 0, 5, 10, 15, and 20 W/g bone, coupled with durations of 10, 15, and 20 min. Exposure time for a modality was varied at 10, 15, and 20 min only when its power was non-zero; when power was 0, its exposure time was fixed at 0 min by design. There were 169 treatment combinations in total. During both ultrasound and microwave treatments, the temperature of the mixture was monitored with a thermocouple (IID, Scientz, Ningbo, China) and fiber-optic sensor (GX-05-4, Wushijin, Nanjing, China) to prevent it from exceeding 60 °C.

Subsequently, 0.25% Protamex (*w*/*w* bone) was added, and the pH was adjusted to 7.0. Enzymatic hydrolysis was conducted at 50 °C for 2.5 h. Afterwards, an additional 0.25% Flavourzyme (*w*/*w* bone) was added, and the pH was adjusted to 7.5, followed by another 2.5 h of enzymatic hydrolysis at 50 °C. Protamex was used to solubilize collagen, followed by Flavourzyme was used to debitter and generate short peptides relevant to antioxidant and aroma functions [54,55]. The reaction was terminated by heating to 90 °C for 15 min to inactivate the enzymes. The enzymatic hydrolysates were centrifuged at 8000× *g* for 15 min at 4 °C and then the supernatant was collected.

### 3.4. Degree of Hydrolysis (DH) Detection

The DH was determined as described by Zheng et al. [4] with minor modifications. 5 mL of the enzymatic hydrolysis solution was measured and diluted 20-fold. 20.00 mL of the diluted solution was transferred into an Erlenmeyer flask, followed by the addition of 60 mL of distilled water. The mixture was then titrated with 0.05 M NaOH until the pH reached 8.2, as indicated by the pH meter. Subsequently, 10 mL of formaldehyde solution was added, ensuring adequate mixing. Titration was then continued with 0.05 M NaOH until the pH meter indicated a pH of 9.2. The volume of NaOH consumed was recorded. For the blank control, 80 mL of ultrapure water was utilized. The total nitrogen content was measured using the Kjeldahl method as described in GB 5009.5 [56]. The *DH* was determined by using Equation (1)

(1) $$DH\left(\%\right)=\frac{(V-V_{0})\times 0.05\times 0.014}{Total\;nitrogen(K=5.79)}\times 100\%,$$

where *V* represents the volume of NaOH consumed following the addition of formaldehyde solution, and *V*_0_ denotes the volume of NaOH used in the blank test.

### 3.5. Back Propagation Artificial Neural Network Mode (BP-ANN) Design

This study employed an ANN to investigate how ultrasound and microwave pre-treatments affect the enzymatic hydrolysis of Xuanwei ham bones. Ultrasound power and time, microwave power and time were selected as the main parameters for the input layer, with DH as the output layer. The number of neurons in the hidden layers varies based on the specific issue being addressed, with consideration given to the potential for model overfitting with too many neurons, and hindered learning capabilities with too few [57]. The range of neurons in the hidden layers was chosen according to Equation (2), and the exact number was determined through trial-and-error method. The BP-ANN was constructed using MATLAB (R2022a).

(2) $$Hiddennum=\sqrt{M+N}+A,$$

where *M* denotes the sample size of input layers. *N* denotes the sample size of output layers and *A* represents any number from 1 to 10.

To address the significant differences in the scale of variables, we standardized the data to a range between 0 and 1 using mean-variance normalization. This preprocessing was carried out prior to model construction, following the transformation outlined in Equation (3).

(3) $$Y=\frac{X_{m}-mean(X_{n})}{\surd var(X_{n})},$$

where *Xm* represents the original data points. *Xn* refers to the series of original data values, and *Y* indicates the data after normalization.

In the model fitting process, a learning rate of 0.01 was set, along with 1000 iterations. The *Relu* function (Equation (4)) and the *Sigmoid* function (Equation (5)) were utilized in the input-to-hidden and hidden-to-output layers, respectively. The application of mean square error (*MSE*) (Equation (6)) facilitated effective parameter optimization in the ANN. The root mean absolute percentage error (*MAPE*) was used to quantify the predictive accuracy of the model on the test set (Equation (7)).

(4) $$Relu(x)=\left\{\begin{array}{c} x,\;\;x\geq 0\\ 0,\;\;x<0 \end{array}\right.,$$

(5) $$Sigmoid(x)=\frac{1}{1+e^{-x}}$$

(6) $$MSE=\frac{1}{n}\sum_{i=1}^{n}(y_{i}-v_{i}{)}^{2}$$

(7) $$MAPE=\frac{1}{n}\sum_{i=1}^{n}\frac{\left|v_{i}-y_{i}\right|}{y_{i}}Relu(x)=\left\{\begin{array}{c} x,\;\;x\geq 0\\ 0,\;\;x<0 \end{array}\right.,$$

where *x* represents the input value, *y_i_* indicates the actual output value, *v_i_* denotes the predicted output value, *n* represents to the sample size.

Ultimately, the experimental datasets were divided into training, validation, and testing subsets, which were then input into the ANN structure. The influence of input layer parameters on the modeled response was assessed by analyzing the distribution of connection weights within the specified ANN topology (Equation (8)) [58].

(8) $$I_{j}=\frac{\sum_{m=1}^{m=N_{h}}((|W_{jm}^{ih}|/\sum_{k=1}^{N_{i}}\left|W_{km}^{ih}|)\times |W_{mn}^{ho}|\right)}{\sum_{k=1}^{k=N_{i}}(\sum_{m=1}^{m=N_{h}}\left(|W_{km}^{ih}|/\sum_{k=1}^{N_{i}}\left|W_{km}^{ih}\right|)\times |W_{mn}^{ho}|\right)}$$

where *I_j_* represents the relative importance of the input factor on the output response. *N_i_* and *N_h_* denote the number of neurons in the input and hidden layers, respectively. *W_s_* refers to the connection weights between layers. The labels *i*, *h* and *o* indicate the input, hidden, and output layers, respectively, while *k*, *m* and *n* specify the neurons in the input, hidden, and output layers, respectively.

ANN is a computational model composed of neurons and their interconnections. Neurons, the basic processing units, are organized into multiple layers and influence each other through weighted connections [59]. Typically, a network includes an input layer, hidden layers, and an output layer. The number of neurons in the hidden layers can vary depending on the complexity of the problem. For this investigation, key parameters selected for the input layer included ultrasound power (0, 5, 10, 15, and 20 W/g bone), ultrasound time (0, 10, 15, and 20 min), microwave power (0, 5, 10, 15, and 20 W/g bone), and microwave time (0, 10, 15, and 20 min). Through single-factor experiments, a dataset comprising 169 groups was utilized for training and predictions using the ANN model.

### 3.6. DPPH Free Radical Scavenging Assay

The free radical scavenging activity was assessed using the DPPH (1,1-diphenyl-2-picrylhydrazyl) assay as described by Huang et al. [60]. An aliquot of 1 mL enzymatic hydrolysate was added to 2 mL of a DPPH methanol solution. The mixture was thoroughly mixed and then kept at 25 °C for 30 min. Subsequently, the absorbance was measured at 517 nm by using a visible spectrophotometer (UV-5100B, Metash Instruments Co. ltd., Shanghai, China).

### 3.7. Thiobarbituric Acid Reactive Substances (TBARS) Value Determination

According to the procedure of Xu et al. [61] with minor modification, a 0.5 g sample of enzymatic hydrolysates was mixed with 3 mL of a stock solution containing 0.375 g/100 mL thiobarbituric acid (TBA) and 15 g/100 mL trichloroacetic acid (TCA) in a 0.25 mol/L HCl solution. This mixture was subsequently heated for 20 min in a boiling water bath to induce the formation of a red color. After heating, the mixture was quickly cooled in an ice bath and centrifuged at 5500× *g* for 15 min. The absorbance of the resulting supernatant was then measured at 532 nm using a spectrophotometer (UV-5100B, Metash Instruments Co. ltd., Shanghai, China).

### 3.8. Gas Chromatography-Mass Spectrometry (GC–MS) Measurement Based on Headspace-Solid Phase Microextraction (HS-SPME)

Volatile compounds from enzymatic hydrolysates were determined using HS-SPME/GC-MS (QP2010, Shimadzu, Kyoto, Japan). Following a slightly modified method reported by Chen, et al. [22], a 5 g sample was placed into a 20 mL headspace vial with 4 µL of 1,2-dichlorobenzene (100 mg/L) added as an internal standard. The vial was sealed with a PTFE silicone septum and equilibrated in a 50 °C water bath for 10 min. Volatile compounds were then extracted with a divinylbenzene/carboxy/polydimethylsiloxane (DVB/CAR/PDMS) fiber (Supelco Inc., Bellefonte, PA, USA) for 30 min at 50 °C. Afterwards, the fiber was desorbed in a GC inlet at 250 °C for 5 min.

A DB-wax capillary column (0.25 mm × 0.25 µm × 30 m) was employed for GC–MS analysis. The oven temperature program commenced at 40 °C, held for 2 min, then increased to 90 °C at a rate of 3 °C/min, maintained for 5 min, escalated to 200 °C at the same rate, and finally reached 230 °C at 15 °C/min, where it was held for 10 min. Helium was used as the carrier gas at a flow rate of 1.5 mL/min, with an interface temperature of 280 °C and an ion source temperature of 230 °C. Electron ionization was set at 70 eV, scanning from 45 to 450 *m*/*z*. Compound identification and quantification were facilitated using the NIST 21 mass spectral library and retention indices, with a similarity threshold above 90% compared against internal standards. Each treatment was replicated three times for accuracy.

The odor activity value (OAV), defined as the ratio of a compound’s concentration to its odor threshold, was used to assess the relative contribution of each volatile to the overall aroma profile.

### 3.9. Electronic Nose (E-Nose) Detection

Following a slightly adjusted method from Xiang et al. [62], the analysis of volatile compounds was conducted using an E-Nose (C-nose, Baosheng, Suining, China) equipped with 18 metal oxide gas sensors (S1–S18). A 4 g sample was positioned in a vial with a 20 mL headspace and stabilized at 50 °C for 50 min. The sensor array was zeroed using processed clean air as the carrier. Subsequently, 200 µL of the headspace was sampled, with a wash time of 100 s and a test duration of 60 s. The device underwent a cleaning cycle lasting 5000 s before each measurement. Each sample was measured three times.

### 3.10. Statistical Analysis

Results were expressed as mean ± standard deviation (SD) and illustrated using Origin2021 Pro software (OriginLab Corporation, Northampton, MA, USA). PCA was performed in Origin on the correlation matrix of the standardized variables, and the PC1–PC2 score and loading plots were generated and exported directly from Origin. The ANN was implemented using MATLAB (R2022a) software. Statistical evaluations were performed with one-way ANOVA and Tukey’s HSD post hoc test, establishing significance at *p* < 0.05.

## 4. Conclusions

In this study, we utilized a BP-ANN to optimize the enzymatic hydrolysis process parameters of Xuanwei ham femur bones, assisted by ultrasound and microwave, and investigated the antioxidant properties and flavor changes in the hydrolysates under optimal conditions. The optimal conditions were determined as 15 w/g bone for ultrasound with 15 min, and 5 w/g bone for microwave with 30 min. Under these conditions, the highest DH was achieved, with the BP-ANN model predicting a DH of 27.69, while the experimentally measured actual DH was 28.33. DPPH radical scavenging and TBARS demonstrated that UMH exhibited the highest antioxidant activity and significantly inhibited lipid oxidation. Furthermore, flavor analysis under UMH conditions revealed substantial changes. Predominantly, there was a removal of bitter volatile flavor compounds, such as o-Cresol and m-Cresol, the retention of aromatic volatile compounds, such as 2-pentylfuran, formation of new aromatic volatile compounds such as 3-methylbutanal, and the reduction in certain aldehyde and ketone compounds. Pearson correlation analysis elucidated that the reduction in aldehyde and ketone compounds was positively linked to the enhanced antioxidant capacity of UMH. In summary, UMH can enhance the DH of Xuanwei ham bones, effectively improving the flavor and antioxidant properties of the enzymatic hydrolysates, increasing the utilization of by-products, and the added value of the Xuanwei ham.

## Figures and Tables

**Table 1:** Optimized ANN architecture, i.e., number of neurons, initialized weight and bias projected.

Neuron Number (j)	Weight to Hidden Layer from Input Variable				To Output Variable
	1	2	3	4	
1	−1.367	−0.472	−0.641	−0.315	0.830
2	−1.982	2.0567	−0.909	−0.548	0.3361
3	2.253	−0.668	0.389	0.347	−0.7664
4	−2.720	−0.049	−0.209	−0.009	−1.198
5	0.833	−0.535	0.667	0.439	0.998
6	2.638	0.457	−1.089	−0.498	0.473

**Table 2:** The BP-ANN predicted and experimental values of DH under different pretreatments. Different letters on the same column indicate significant differences (*p* < 0.05).

Experiment Number	Ultrasound		Microwave		DH (%)	
	Power (W/g_bone_)	Time (min)	Power (W/g_bone_)	Time (min)	Expt.	BP-ANN Pred.
1	15	15	5	20	26.65 ± 0.20 ^b^	26.15
2	15	15	5	30	28.33 ± 0.18 ^a^	27.69
3	15	15	7	20	27.05 ± 0.63 ^b^	26.49
4	15	15	7	30	28.16 ± 0.42 ^a^	27.53
5	20	15	5	30	26.05 ± 0.32 ^c^	25.63
6	20	15	7	30	25.21 ± 0.10 ^c^	25.49

**Table 3:** Volatile compounds with OAV > 1 in Xuanwei ham bone enzymatic hydrolysates with different pretreatments. ND indicates that the substance was not detected. (BBC: Bone broth control; HBH: Ham bone hydrolysates; MWH: Microwave pretreatment hydrolysates; UFH: Ultrasound pretreatment hydrolysates; UMH: Ultrasound combined microwave pretreatment hydrolysates).

Volatile Compounds	Threshold (μg/kg)	BBC	HBH	MWH	UFH	UMH
p-Cresol	0.00024	ND	ND	250.16	ND	ND
m-Cresol	0.00044	ND	ND	ND	95.35	ND
4-Ethylphenol	0.021	11.81	11.81	ND	ND	2.43
2-Pentylfuran	0.27	12.78	5.37	9.40	6.38	11.46
3-Methylbutanal	0.00035	ND	ND	ND	66.94	62.80
Pentanal	0.85	1.75	0.88	0.35	0.39	0.40
Hexanal	5	13.27	4.92	2.72	2.62	3.25
Heptanal	0.26	11.57	4.23	3.28	ND	ND
Octanal	0.00088	2587.01	3181.82	1955.86	453.44	678.04
(E)-2-Heptenal	2.4	1.56	0.88	0.80	0.53	0.78
Nonanal	0.0031	799.29	451.61	597.00	199.66	230.34
(E)-2-Octenal	0.0027	2236.00	1111.11	1113.32	667.38	767.18
(E, E)-2,4-Heptadienal	0.057	2.81	3.16	ND	2.88	0.70
(E)-2-Nonenal	0.00009	17,402.65	12,222.22	9352.11	4184.33	4964.80
(E)-2-Decenal	0.0027	620.61	481.48	437.22	114.68	111.71
(E, E)-2,4-Nonadienal	0.0002	1910.11	1050.00	922.96	655.56	3074.36
(E)-2-Dodecenal	0.011	103.27	90.91	68.01	13.06	ND
(E, E)-2,4-Decadienal	0.0023	702.85	491.30	210.15	103.63	114.61
2-Methylenehexanal	0.1	2.98	ND	ND	0.43	0.74
(E)-2-Hexenal	0.0031	123.54	ND	ND	ND	50.52
Decanal	0.0026	54.63	ND	ND	ND	ND
2-Heptanone	0.0035	554.92	228.57	186.37	ND	ND
6-Methylhept-5-en-2-one	0.01889	ND	3.71	2.88	2.00	2.60
3-Octen-2-one	0.0067	1273.34	671.64	437.67	414.55	542.94

## Data Availability

The raw data supporting the conclusions of this article will be made available by the authors on request.

## Supplementary Materials

The following supporting information can be downloaded at: https://www.mdpi.com/article/10.3390/molecules31010188/s1, Table S1: Degree of hydrolysis of enzymatic hydrolysate under different pretreatments; Table S2: Volatile compounds in Xuanwei ham bone enzymatic hydrolysate with different pretreatments.

## Abbreviations

The following abbreviations are used in this manuscript:

E-Nose	Electronic Nose
BBC	Bone Broth Control
DH	Degree of Hydrolysis
GC–MS	Gas Chromatography-Mass Spectrometry
HBH	Ham Bone Hydrolysates
HS-SPME	Headspace-Solid Phase Microextraction
MWH	Microwave Pretreatment Hydrolysates
TBARS	Thiobarbituric Acid Reactive Substances
UFH	Ultrasound Pretreatment Hydrolysates
UMH	Ultrasound Combined Microwave Pretreatment Hydrolysates

## References

[1] Cui H., Li H., Wu Y., Hu X. (2023). Identification, flavor characteristics and molecular docking of umami taste peptides of Xuanwei ham. Food Res. Int..

[2] Gallego M., Mora L., Hayes M., Reig M., Toldrá F. (2019). Peptides with Potential Cardioprotective Effects Derived from Dry-Cured Ham Byproducts. J. Agric. Food Chem..

[3] Zheng Z., Zhang M., Liu W., Liu Y. (2022). Effect of beef tallow, phospholipid and microwave combined ultrasonic pretreatment on Maillard reaction of bovine bone enzymatic hydrolysate. Food Chem..

[4] Zheng Z., Zhang M., Fan H., Liu Y. (2021). Effect of microwave combined with ultrasonic pretreatment on flavor and antioxidant activity of hydrolysates based on enzymatic hydrolysis of bovine bone. Food Biosci..

[5] Yao Y., Wang M., Liu Y., Han L., Liu X. (2020). Insights into the improvement of the enzymatic hydrolysis of bovine bone protein using lipase pretreatment. Food Chem..

[6] Wang Y., Tang X., Luan J., Zhu W., Xu Y., Yi S., Li J., Wang J., Li X. (2022). Effects of ultrasound pretreatment at different powers on flavor characteristics of enzymatic hydrolysates of cod (*Gadus macrocephalus*) head. Food Res. Int..

[7] Zhou S., Chen W., Fan K. (2024). Recent advances in combined ultrasound and microwave treatment for improving food processing efficiency and quality: A review. Food Biosci..

[8] Zhu Y., Zhang M., Law C.L., Wang Y., Liu K. (2023). Optimization of Ultrasonic-Assisted Enzymatic Hydrolysis to Extract Soluble Substances from Edible Fungi By-products. Food Bioprocess Technol..

[9] Liu F., Li Y., Sun G., Wang C., Liang Y., Zhao X., He J., Mo H. (2022). Influence of ultrasound treatment on the physicochemical and antioxidant properties of mung bean protein hydrolysate. Ultrason. Sonochem..

[10] Huang S., Li Y., Li C., Ruan S., Roknul Azam S.M., Ou Yang N., Ye X., Wang Y., Ma H. (2021). Effects of ultrasound-assisted sodium bisulfite pretreatment on the preparation of cholesterol-lowering peptide precursors from soybean protein. Int. J. Biol. Macromol..

[11] Suhag R., Dhiman A., Deswal G., Thakur D., Sharanagat V.S., Kumar K., Kumar V. (2021). Microwave processing: A way to reduce the anti-nutritional factors (ANFs) in food grains. LWT-Food Sci. Technol..

[12] Habinshuti I., Mu T.H., Zhang M. (2020). Ultrasound microwave-assisted enzymatic production and characterisation of antioxidant peptides from sweet potato protein. Ultrason. Sonochem..

[13] Huang Y., Ruan G., Qin Z., Li H., Zheng Y. (2017). Antioxidant activity measurement and potential antioxidant peptides exploration from hydrolysates of novel continuous microwave-assisted enzymolysis of the Scomberomorus niphonius protein. Food Chem..

[14] Huang X., You Y., Zeng X., Liu Q., Dong H., Qian M., Xiao S., Yu L., Hu X. (2024). Back propagation artificial neural network (BP-ANN) for prediction of the quality of gamma-irradiated smoked bacon. Food Chem..

[15] Lin Y., Ma J., Wang Q., Sun D. (2023). Applications of machine learning techniques for enhancing nondestructive food quality and safety detection. Crit. Rev. Food Sci. Nutr..

[16] Jiao X., Ren G., Law C.L., Li L., Cao W., Luo Z., Pan L., Duan X., Chen J., Liu W. (2024). Novel strategy for optimizing of corn starch-based ink food 3D printing process: Printability prediction based on BP-ANN model. Int. J. Biol. Macromol..

[17] Han Z., Gao J. (2019). Pixel-level aflatoxin detecting based on deep learning and hyperspectral imaging. Comput. Electron. Agric..

[18] Liu Y., Gong H., Shi C., Yuan H., Zuo X., Chang Y., Li X. (2022). Modeling and optimization of the hydrolysis and acidification via liquid fraction of digestate from corn straw by response surface methodology and artificial neural network. J. Clean. Prod..

[19] Li S., Hu Y., Hong Y., Xu L., Zhou M., Fu C., Wang C., Xu N., Li D. (2016). Analysis of the hydrolytic capacities of Aspergillus oryzae proteases on soybean protein using artificial neural networks. J. Food Process. Preserv..

[20] Wang C., Shi X., Xue J., Zhao S., Jia C., Niu M., Zhang B., Xu Y. (2024). Quality prediction of whole-grain rice noodles using backpropagation artificial neural network. J. Sci. Food Agric..

[21] Bhagya Raj G.V.S., Dash K.K. (2022). Comprehensive study on applications of artificial neural network in food process modeling. Crit. Rev. Food Sci. Nutr..

[22] Chen Y., Cai K., Tu Z., Nie W., Ji T., Hu B., Chen C., Jiang S. (2018). Prediction of benzo[a]pyrene content of smoked sausage using back-propagation artificial neural network. J. Sci. Food Agric..

[23] Tang P.L., Koh X.J. (2023). Ultrasound-assisted enzymatic hydrolysis enhances anti-inflammatory and hypoglycemic activities of edible Bird’s nest. Food Biosci..

[24] Ayim I., Ma H., Alenyorege E.A., Ali Z., Donkor P.O. (2018). Influence of ultrasound pretreatment on enzymolysis kinetics and thermodynamics of sodium hydroxide extracted proteins from tea residue. J. Food Sci. Technol..

[25] Yan J., Pei J., Ma H., Wang Z. (2015). Effects of ultrasound on molecular properties, structure, chain conformation and degradation kinetics of carboxylic curdlan. Carbohydr. Polym..

[26] Pi X., Liu J., Ren S., Zhu L., Li B., Zhang B. (2024). Research progress in ultrasound and its assistance treatment to reduce food allergenicity: Mechanisms, influence factor, application and prospect. Int. J. Biol. Macromol..

[27] Rahman M.M., Lamsal B.P. (2021). Ultrasound-assisted extraction and modification of plant-based proteins: Impact on physicochemical, functional, and nutritional properties. Compr. Rev. Food Sci. Food Saf..

[28] Wang D., Yan L., Ma X., Wang W., Zou M., Zhong J., Ding T., Ye X., Liu D. (2018). Ultrasound promotes enzymatic reactions by acting on different targets: Enzymes, substrates and enzymatic reaction systems. Int. J. Biol. Macromol..

[29] Abdualrahman M.A.Y., Ma H., Zhou C., Yagoub A.E.A., Hu J., Yang X. (2016). Thermal and single frequency counter-current ultrasound pretreatments of sodium caseinate: Enzymolysis kinetics and thermodynamics, amino acids composition, molecular weight distribution and antioxidant peptides. J. Sci. Food Agric..

[30] Wali A., Ma H., Hayat K., Ren X., Ali Z., Duan Y., Rashid M.T. (2018). Enzymolysis reaction kinetics and thermodynamics of rapeseed protein with sequential dual-frequency ultrasound pretreatment. Int. J. Food Sci. Technol..

[31] Hall F., Liceaga A. (2020). Effect of microwave-assisted enzymatic hydrolysis of cricket (*Gryllodes sigillatus*) protein on ACE and DPP-IV inhibition and tropomyosin-IgG binding. J. Funct. Foods.

[32] Li Y., Li J., Lin S., Yang Z., Jin H. (2019). Preparation of Antioxidant Peptide by Microwave- Assisted Hydrolysis of Collagen and Its Protective Effect Against H_2_O_2_-Induced Damage of RAW264.7 Cells. Mar. Drugs.

[33] Kang D.C., Zou Y.H., Cheng Y.P., Xing L.J., Zhou G.H., Zhang W.G. (2016). Effects of power ultrasound on oxidation and structure of beef proteins during curing processing. Ultrason. Sonochem..

[34] Tawalbeh D., Al-U’datt M.H., Wan Ahmad W.A., Ahmad F., Sarbon N.M. (2023). Recent Advances in In Vitro and In Vivo Studies of Antioxidant, ACE-Inhibitory and Anti-Inflammatory Peptides from Legume Protein Hydrolysates. Molecules.

[35] Zheng L., Zhao Y., Dong H., Su G., Zhao M. (2016). Structure–activity relationship of antioxidant dipeptides: Dominant role of Tyr, Trp, Cys and Met residues. J. Funct. Foods.

[36] Xu P., Zheng Y., Zhu X., Li S., Zhou C. (2018). L-lysine and L-arginine inhibit the oxidation of lipids and proteins of emulsion sausage by chelating iron ion and scavenging radical. Asian-Australas. J. Anim. Sci..

[37] Xu Y., Yang Y., Ma C.M., Bian X., Liu X.F., Wang Y., Chen F.L., Wang B., Zhang G., Zhang N. (2023). Characterization of the structure, antioxidant activity and hypoglycemic activity of soy (*Glycine max* L.) protein hydrolysates. Food Res. Int..

[38] Ketnawa S., Liceaga A.M. (2017). Effect of Microwave Treatments on Antioxidant Activity and Antigenicity of Fish Frame Protein Hydrolysates. Food Bioprocess Technol..

[39] Lone A.B., Bhat H.F., AïtKaddour A., Hassoun A., Aadil R.M., Dar B.N., Bhat Z.F. (2023). Cricket protein hydrolysates pre-processed with ultrasonication and microwave improved storage stability of goat meat emulsion. Innov. Food Sci. Emerg. Technol..

[40] Peng Z., Wang F., Yu L., Jiang B., Cao J., Sun Z., Cheng J. (2024). Effect of ultrasound on the characterization and peptidomics of foxtail millet bran protein hydrolysates. Ultrason. Sonochem..

[41] Sinthusamran S., Benjakul S., Kijroongrojana K., Prodpran T., Agustini T.W. (2018). Yield and chemical composition of lipids extracted from solid residues of protein hydrolysis of Pacific white shrimp cephalothorax using ultrasound-assisted extraction. Food Biosci..

[42] Li X., Liu Y., Wang Y., Wang J., Xu Y., Yi S., Zhu W., Mi H., Li T., Li J. (2021). Combined ultrasound and heat pretreatment improve the enzymatic hydrolysis of clam (*Aloididae aloidi*) and the flavor of hydrolysates. Innov. Food Sci. Emerg. Technol..

[43] Huang Y., Kao T., Chen B. (2022). Development of a GC–MS/MS method coupled with HS-SPME-Arrow for studying formation of furan and 10 derivatives in model systems and commercial foods. Food Chem..

[44] He W., Liu Z., Liu H., Sun J., Chen H., Sun B. (2024). Characterization of key volatile flavor compounds in dried sausages by HS-SPME and SAFE, which combined with GC-MS, GC-O and OAV. J. Food Compos. Anal..

[45] Zheng Z., Zhang L., Zhang M., Mujumdar A.S., Liu Y. (2023). Maillard reaction products of pea protein hydrolysate as a flavour enhancer for beef flavors: Effects on flavor and physicochemical properties. Food Chem..

[46] Czelej M., Garbacz K., Czernecki T., Wawrzykowski J., Waśko A. (2022). Protein Hydrolysates Derived from Animals and Plants—A Review of Production Methods and Antioxidant Activity. Foods.

[47] Acquaticci L., Schouten M.A., Angeloni S., Caprioli G., Vittori S., Romani S. (2024). Influence of baking conditions and formulation on furanic derivatives, 3-methylbutanal and hexanal and other quality characteristics of lab-made and commercial biscuits. Food Chem..

[48] Zhang J., Mora L., Toldrá F., Zhang W., Flores M. (2024). Effects of ultrasound pretreatment on flavor characteristics and physicochemical properties of dry-cured ham slices during refrigerated vacuum storage. LWT-Food Sci. Technol..

[49] Grebenteuch S., Kroh L.W., Drusch S., Rohn S. (2021). Formation of Secondary and Tertiary Volatile Compounds Resulting from the Lipid Oxidation of Rapeseed Oil. Foods.

[50] Zhang Q., Ke J., Long P., Wen M., Han Z., Zhang L., Zhu M. (2024). Formation of Furan from Linoleic Acid Thermal Oxidation: (E,E)-2,4-Decadienal as a Critical Intermediate Product. J. Agric. Food Chem..

[51] Feng Y., Cai Y., Fu X., Zheng L., Xiao Z., Zhao M. (2018). Comparison of aroma-active compounds in broiler broth and native chicken broth by aroma extract dilution analysis (AEDA), odor activity value (OAV) and omission experiment. Food Chem..

[52] Yasunaga M., Takai E., Hattori S., Tatematsu K., Kuroda S.i. (2022). Effects of 3-octen-2-one on human olfactory receptor responses to vanilla flavor. Biosci. Biotechnol. Biochem..

[53] Xi Y., Ikram S., Zhao T., Shao Y., Liu R., Song F., Sun B., Ai N. (2023). 2-Heptanone, 2-nonanone, and 2-undecanone confer oxidation off-flavor in cow milk storage. J. Dairy Sci..

[54] Hong G., Min S., Jo Y. (2019). Anti-Oxidative and Anti-Aging Activities of Porcine By-Product Collagen Hydrolysates Produced by Commercial Proteases: Effect of Hydrolysis and Ultrafiltration. Molecules.

[55] Merz M., Eisele T., Berends P., Appel D., Rabe S., Blank I., Stressler T., Fischer L. (2015). Flavourzyme, an Enzyme Preparation with Industrial Relevance: Automated Nine-Step Purification and Partial Characterization of Eight Enzymes. J. Agric. Food Chem..

[56] (2016). Determination of Protein in Food.

[57] Cervera Gascó J., Rabadán A., López Mata E., Álvarez Ortí M., Pardo J.E. (2023). Development of the POLIVAR model using neural networks as a tool to predict and identify monovarietal olive oils. Food Control.

[58] Garson D.G. (1991). Interpreting neural network connection weights. Al Expert.

[59] Kothakota A., Pandiselvam R., Siliveru K., Pandey J.P., Sagarika N., Srinivas C.H.S., Kumar A., Singh A., Prakash S.D. (2021). Modeling and Optimization of Process Parameters for Nutritional Enhancement in Enzymatic Milled Rice by Multiple Linear Regression (MLR) and Artificial Neural Network (ANN). Foods.

[60] Huang J., Chen C., Song Z., Chang M., Yao L., Jin Q., Wang X. (2022). Effect of microwave pretreatment of perilla seeds on minor bioactive components content and oxidative stability of oil. Food Chem..

[61] Xu M., Liu Q., Ni X., Chen C., Deng X., Fang Y., Wang X., Shen Q., Yu R. (2024). Lipidomics reveals the effect of hot-air drying on the quality characteristics and lipid oxidation of Tai Lake whitebait (*Neosalanx taihuensis* Chen). LWT-Food Sci. Technol..

[62] Xiang J., Wang X., Guo C., Zang L., He H., Yin X., Wei J., Cao J. (2024). Quality and Flavor Difference in Dry-Cured Meat Treated with Low-Sodium Salts: An Emphasis on Magnesium. Molecules.

