# Supplementary figures and images for: Enhancing Antioxidant and Flavor of Xuanwei Ham Bone Hydrolysates via Ultrasound and Microwave Pretreatment: A Backpropagation Artificial Neural Network Model Prediction
Source: Molecules. 2026 Jan 4;31(1):188. doi: 10.3390/molecules31010188 (PMC12787840; doi:10.3390/molecules31010188)

**A**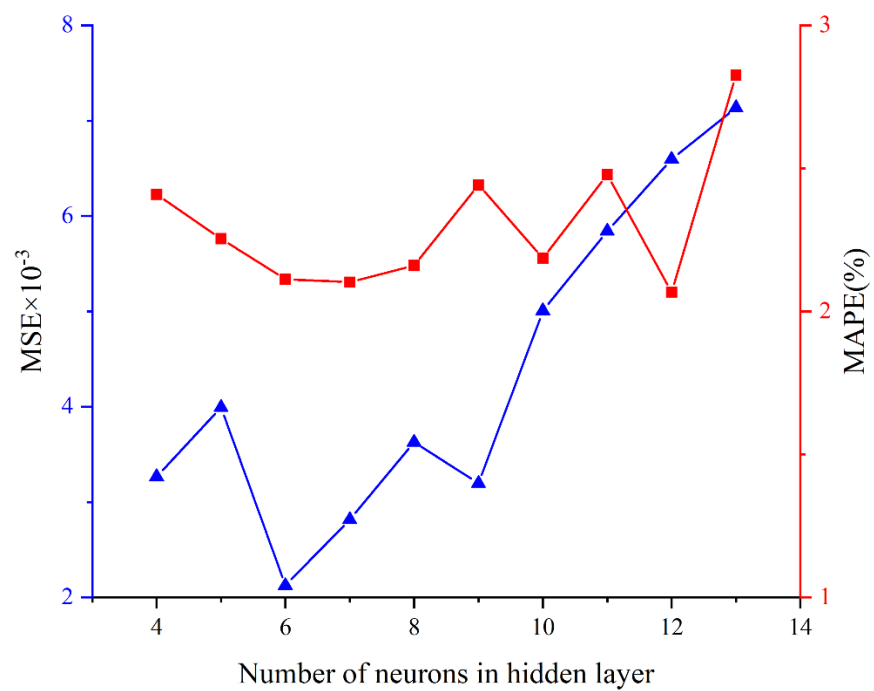**B**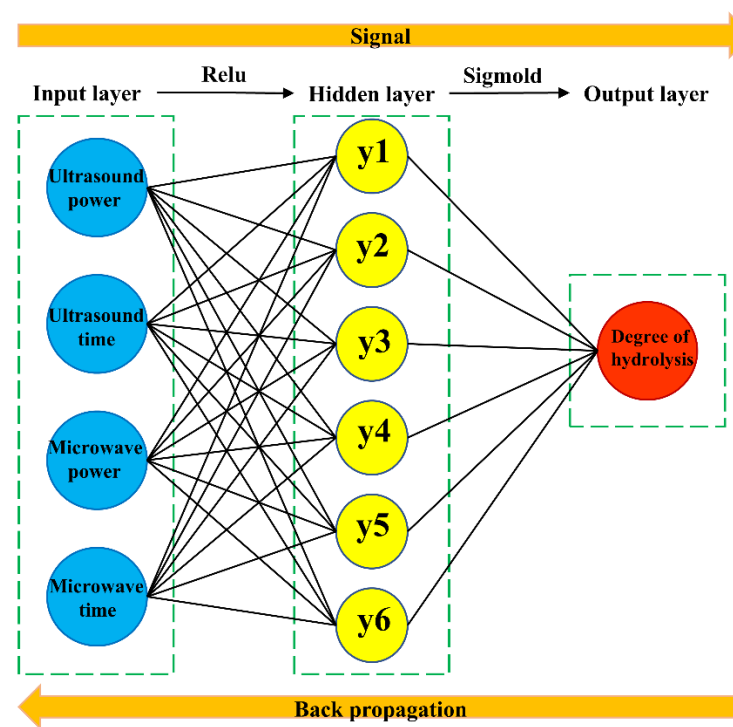

Figure 1

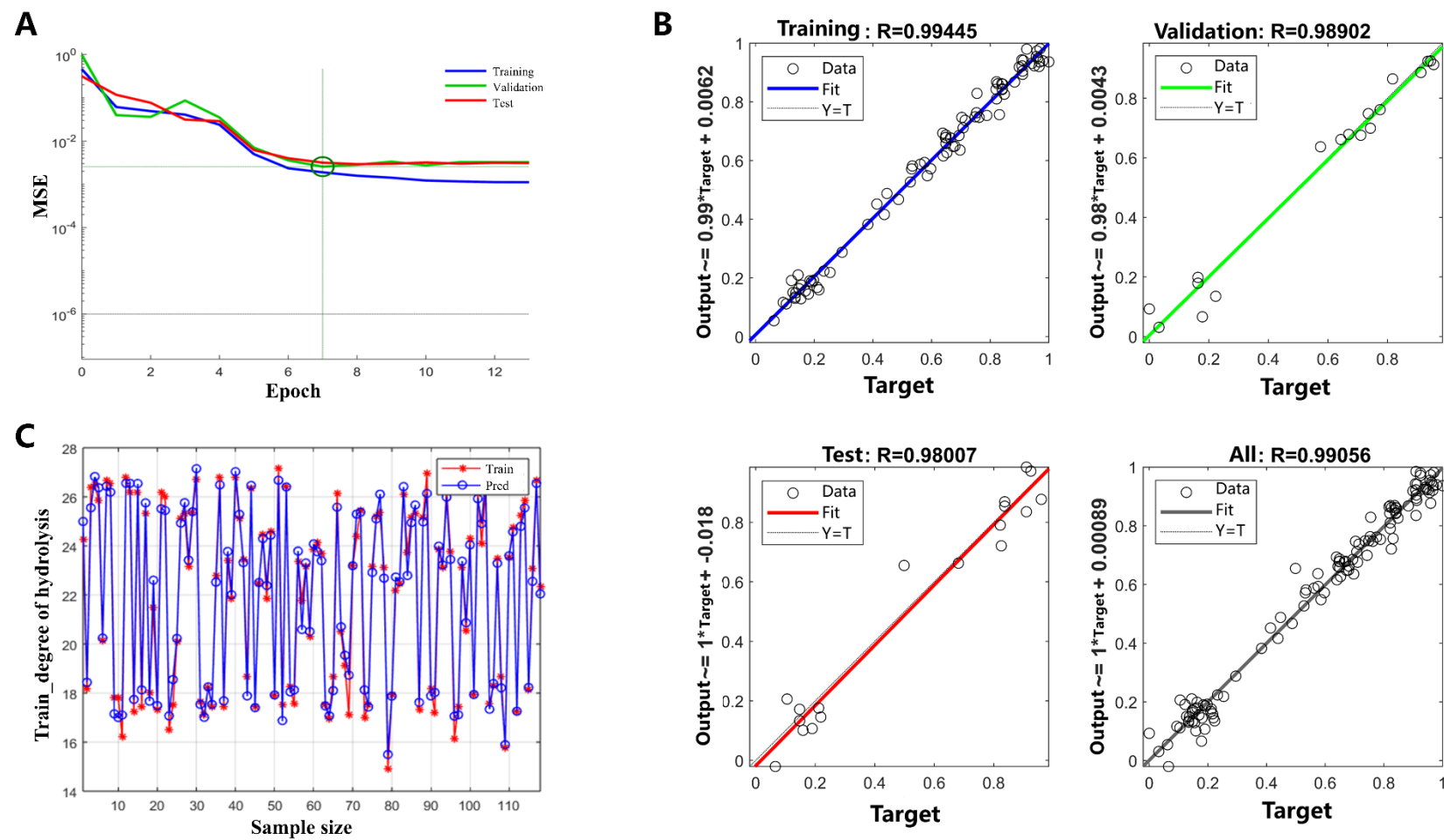

Figure 2

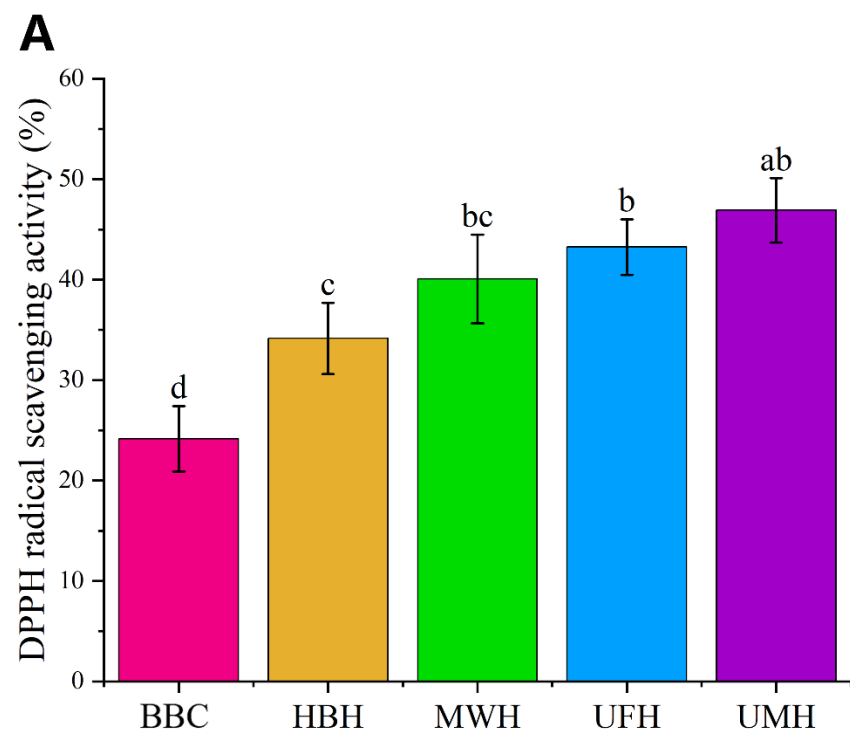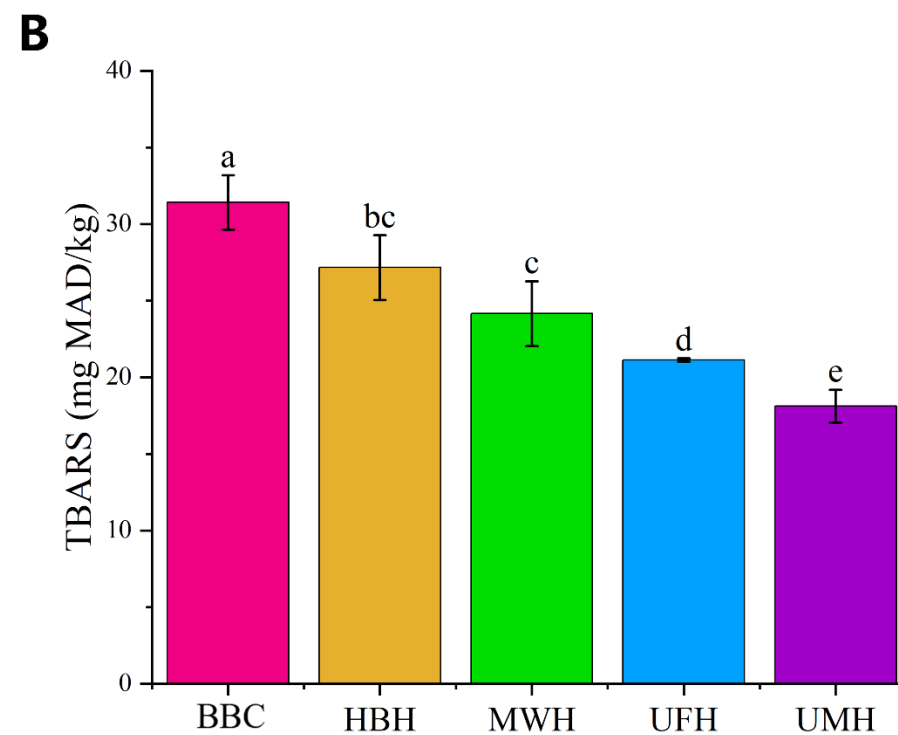

**Figure 3**

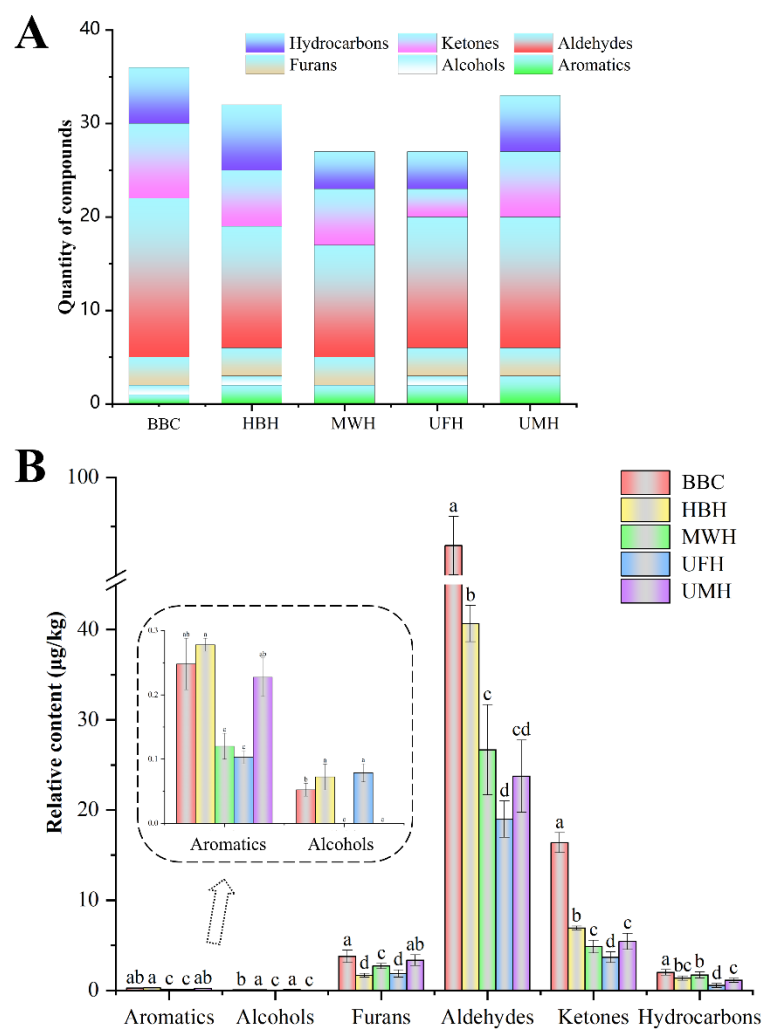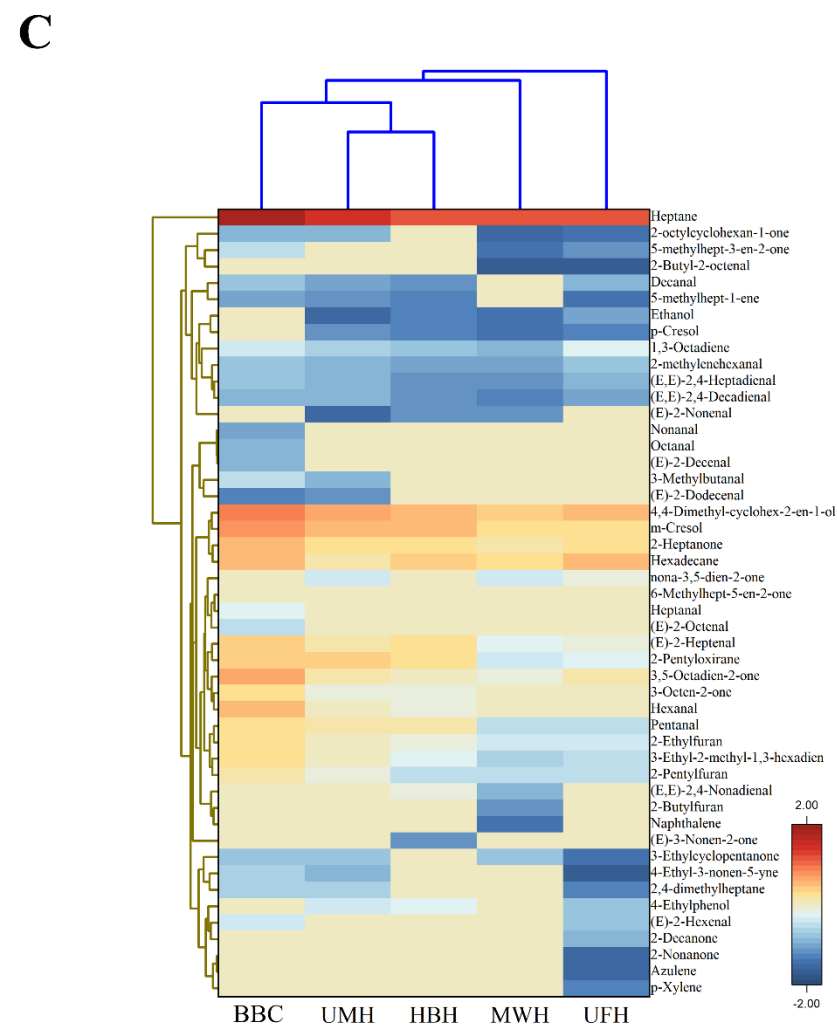

Figure 4

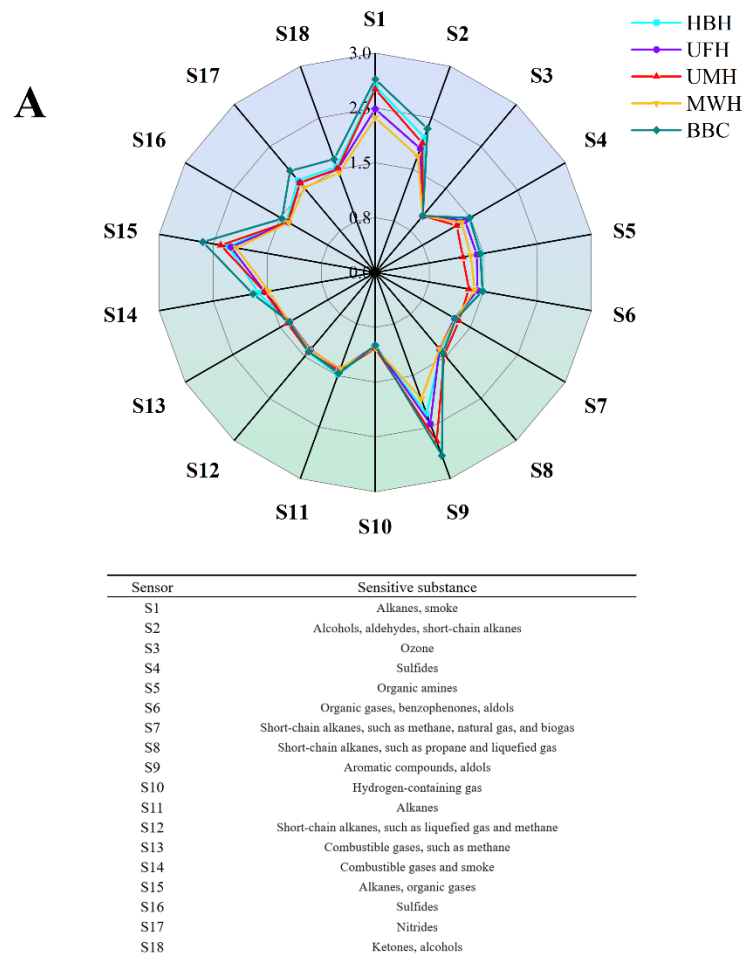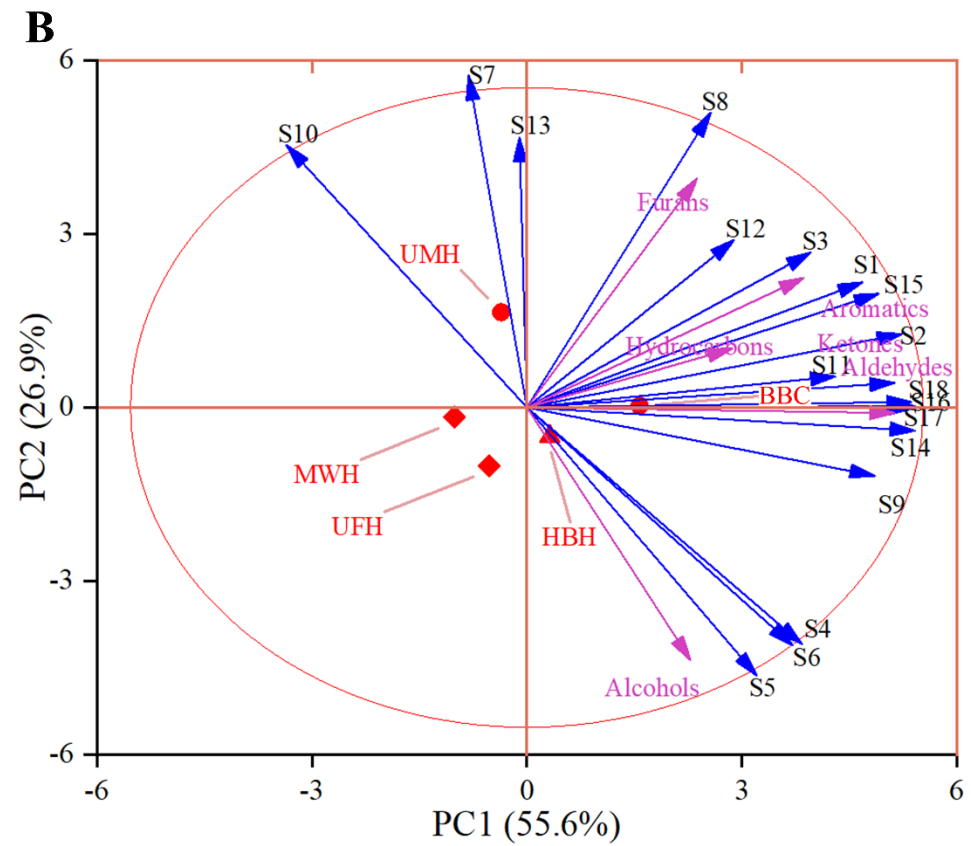

Figure 5

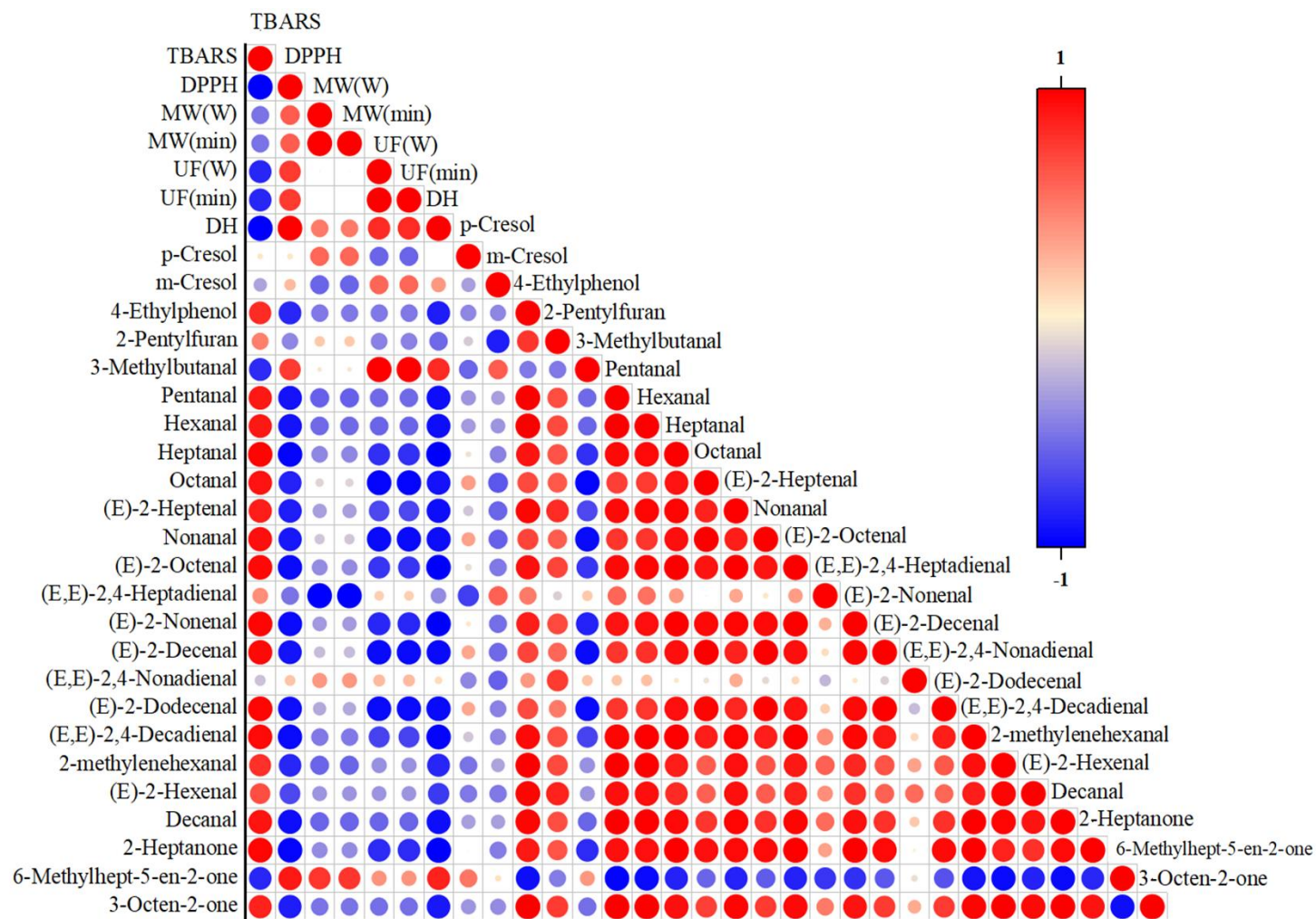

Figure 6

Supplement: Supplementary file 1 [file molecules-31-00188-s001.zip › molecules-4047286-Supplementary Materials.pdf]
